# Photoacoustic-based visual servoing of a needle tip

**DOI:** 10.1038/s41598-018-33931-9

**Published:** 2018-10-19

**Authors:** Muyinatu A. Lediju Bell, Joshua Shubert

**Affiliations:** 1 Johns Hopkins University, Department of Electrical and Computer Engineering, Baltimore, MD 21218 USA; 2 Johns Hopkins University, Department of Biomedical Engineering, Baltimore, MD 21218 USA; 3 Johns Hopkins University, Department of Computer Science, Baltimore, MD 21218 USA

## Abstract

In intraoperative settings, the presence of acoustic clutter and reflection artifacts from metallic surgical tools often reduces the effectiveness of ultrasound imaging and complicates the localization of surgical tool tips. We propose an alternative approach for tool tracking and navigation in these challenging acoustic environments by augmenting ultrasound systems with a light source (to perform photoacoustic imaging) and a robot (to autonomously and robustly follow a surgical tool regardless of the tissue medium). The robotically controlled ultrasound probe continuously visualizes the location of the tool tip by segmenting and tracking photoacoustic signals generated from an optical fiber inside the tool. System validation in the presence of fat, muscle, brain, skull, and liver tissue with and without the presence of an additional clutter layer resulted in mean signal tracking errors <2 mm, mean probe centering errors <1 mm, and successful recovery from ultrasound perturbations, representing either patient motion or switching from photoacoustic images to ultrasound images to search for a target of interest. A detailed analysis of channel SNR in controlled experiments with and without significant acoustic clutter revealed that the detection of a needle tip is possible with photoacoustic imaging, particularly in cases where ultrasound imaging traditionally fails. Results show promise for guiding surgeries and procedures in acoustically challenging environments with this novel robotic and photoacoustic system combination.

## Introduction

Surgical and interventional procedures often require visualization and tracking of needle, catheter, and other tool tips in order to determine their relative locations with respect to a target of interest. Ultrasound is one of the most ideal real-time imaging options available to accomplish this task^1,2^ in soft tissue organs like the liver^3^, brain^4^, and breast^5^, given the technique’s portability, low cost, and high frame rates. In addition, ultrasound navigation does not require harmful ionizing radiation, unlike x-ray fluoroscopy, nor injection of contrast agents, unlike angiography. Despite these advantages, ultrasound imaging often fails in acoustically challenging environments, characterized by significant sound scattering, sound attenuation, and acoustic clutter. Examples include imaging through the skull^6^ or imaging through multiple layers of near-field tissue^7–11^, such as tissue within the abdominal wall^12^.

For example, when imaging through the skull, which causes high acoustic impedance mismatches because the speed of sound and density of bone are significantly larger than those of surrounding soft tissues, the two-way sound penetration through bone is often limited to a few millimeters. Specific requirements for suitable bone thicknesses depend on the ultrasound transducer wavelength, with lower frequencies required for greater acoustic penetration^13,14^. As a trade-off, while lower frequencies improve penetration, they result in images with reduced spatial resolution. Thus, when imaging through the skull, the presence of cranial bone complicates traditional ultrasound guidance of minimally invasive neurosurgery.

Similarly, acoustic clutter^12^ is a common challenge when imaging overweight or obese patients, who represent over 37% of adults in the United States^15,16^. In these patients, sound can be scattered by multiple tissue layers (i.e., multipath scattering), resulting in reduced ultrasound image quality, which is additionally degraded when one or more surgical instruments obscure instrument location, orientation, geometry, and nearby tissues^17^. It is often more difficult to visualize the needle tip in the presence of acoustic clutter, which results in multiple needle passes to obtain an adequate biopsy sample, and the multiple needle passes can cause serious complications, like intraperitoneal hemorrhaging^18,19^. This challenge is particularly present in obese patients, who are considered to be high-risk patients for ultrasound-guided percutaneous liver and kidney biopsies, where a needle is inserted through the skin^10,18,19^.

Tool tip visualization is additionally complicated with traditional ultrasound imaging because it is often difficult to distinguish a tool tip from a similarly appearing tool midsection. While 3D ultrasound imaging may be used to visualize more of the tool body^2,20^, segmenting the tool tip from the image volume remains as a challenge due to multiple image artifacts^17,20^. As an alternative, active tracking of the tool position and orientation can be implemented with sensors. Optical sensors provide highly accurate tracking information, but they require the addition of optical markers, cameras, and a continuous line of sight to the markers, which complicates direct tracking of the tool tip when inserted in the patient^21^. Electromagnetic sensors track tools within the body, but they are less accurate and prone to distortion and noise, particularly in the presence of metallic tools^22^. Both passive and active ultrasound markers have been added to tool tips to enable detection of the tool tip directly in the ultrasound image^23–26^, but passive markers are not visible in acoustically challenging environments and active markers are not effective when the active element is outside of the ultrasound image plane.

Photoacoustic imaging is a promising alternative to visualize the tips of needles, catheters, and other surgical tools. This imaging method uses pulsed laser light to incite thermal expansion, which generates acoustic signals that can be detected with conventional ultrasound transducers^27–29^. The relative intensities of these signals (i.e., image contrast) is determined by the wavelength-dependent optical absorption of the imaged region. For example, blood and metal tend to have higher optical absorption than surrounding tissue, which makes them excellent targets for photoacoustic imaging. When tuned to different wavelengths, photoacoustic imaging may also be used to distinguish blood from lipid-rich structures, such as plaque in blood vessels^30^ or the myelin sheath of nerves^31,32^. As a result, photoacoustic imaging has demonstrated potential to detect metal implants^33–35^, monitor vessel flow^36^, map vessel structure^37^, determine the presence of atherosclerosis^38^, and guide minimally invasive surgeries^39–45^.

When guiding surgeries with photoacoustic imaging, acoustic signals that are concentrated at the tip of a surgical tool can be generated when one end of an optical fiber is connected to a pulsed laser source and the other end is either inserted into the tool^42^ or externally attached to the tool with light directed toward the tool tip^41,44^. The resulting photoacoustic images enable visualization and localization of the tool tip when it is in the image plane. The photoacoustic images can also be used to visualize the separation between critical blood vessels or nerves that are in the vicinity of the surgical tool^40^. While photoacoustic imaging has demonstrated promise to address the challenge of tool tip visualization during surgery, this new approach to photoacoustic imaging requires additional coordination of the required imaging system components (i.e., optical fibers attached to surgical tools that are separated from the ultrasound probe).

We are exploring the feasibility of incorporating assistance from robots to facilitate photoacoustic image guidance. Previous work demonstrated photoacoustic imaging system integration with teleoperated control of the imaging system components with a da Vinci surgical robot^40,44,46^. Building on the existence of several robotic platforms that use visual servoing to autonomously maintain sight of an ultrasound imaging target^47–49^, we developed a novel robotic platform to perform intraoperative catheter, needle, or surgical tool tracking and guidance in acoustically challenging environments. Our platform combines photoacoustic imaging (to improve the visualization of the tool tips) with a servoing robot that continuously maintains sight of the tool tip. The potential benefits of the proposed robotic assistant includes freeing at least one hand of the operator to perform more essential tasks (and in some cases freeing the time of a second operator who may be tasked with manually holding an ultrasound probe and following the tip of a needle, catheter, or surgical tool during insertion), reducing the mental effort and cognitive load that is required to search for the tip with a handheld ultrasound probe (which can reduce errors during procedures^50^), and possibly enabling autonomous surgical and biopsy tasks in the future.

By combining the localized signal generation of photoacoustic imaging and the hands-free visualization provided by a robotic assistant, we propose and investigate a novel approach to interventional guidance in acoustically challenging environments. This approach was originally introduced in our associated conference paper^51^ that describes a robotic photoacoustic system to improve liver biopsy in obese patients, where we investigated system performance in the presence of fat, muscle, and liver tissue, as these are the tissue types that would be encountered during percutaneous liver biopsies. In this paper, we expand our work to include an assessment of photoacoustic channel data under various noise conditions and to explore the performance of our system in the presence of additional tissue types, such as bone and brain tissues. We also characterize photoacoustic image quality as a function of multiple laser energy levels in these multiple tissue types.

## Results

### Impact of Acoustic Clutter

**Figure 1:**
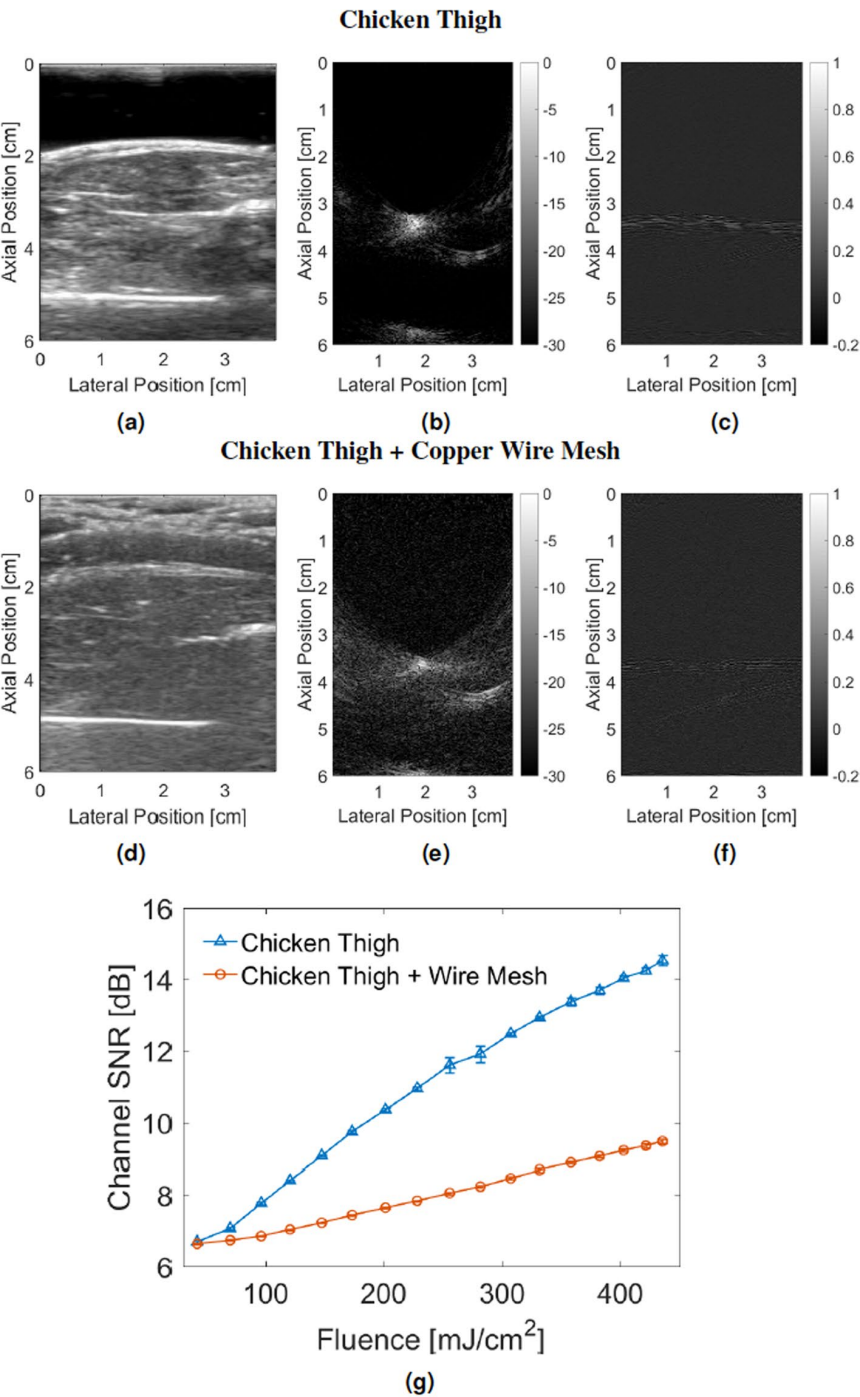
Effect of acoustic clutter demonstrated with (**a**) ultrasound image of an *ex vivo* chicken thigh with a needle inserted and a water bag used as a standoff material, (**b**) corresponding photoacoustic image, and (**c**) corresponding photoacoustic channel data compared to (**d**) ultrasound image of the same setup in the presence of a clutter generating layer consisting of a water bag filled with a wire mesh, (**e**) corresponding photoacoustic image, and (**f**) corresponding photoacoustic channel data generated in the presence of the added clutter layer. The photoacoustic images were acquired with 1.58 mJ pulse energy (i.e., 201.2 mJ/cm^2^ fluence) and are shown on a log scale (30 dB dynamic range), while the images of the channel data are shown on a linear scale (after normalization to the brightest pixel in the image). (**g**) Mean channel SNR of ten acquisitions as a function of incident laser fluence, with error bars showing ± one standard deviation of the ten measurements.

Representative ultrasound images in the absence and presence of a clutter-generating wire mesh are shown in Fig. 1(a,d), respectively. In the ultrasound image acquired with the wire mesh in front of the transducer face (Fig. 1(d)), the needle is not visible due to significant acoustic clutter. The ultrasound probe and *ex vivo* chicken thigh sample remained in the same positions for both acquisitions.

The corresponding photoacoustic images in the absence and presence of the clutter-generating wire mesh are shown in Fig. 1(b,e), respectively. Although, the needle tip was not visible in the cluttered ultrasound image, it appears in the corresponding photoacoustic image of Fig. 1(e), albeit with reduced amplitude when compared to the corresponding photoacoustic image acquired without the wire mesh present (Fig. 1(b)).

The time-delayed photoacoustic channel data for a central lateral line that intersects the photoacoustic signal in the beamformed image is displayed in the absence (Fig. 1(c)) and presence (Fig. 1(f)) of the clutter-generating wire mesh. This display format lends insight into the reasons for the differences in appearance between the photoacoustic images of the needle tip acquired with and without the clutter-generating wire mesh. The channel data shows significant degradation of the raw photoacoustic signals, which is the primary reason for the lower amplitude in the photoacoustic image. This lower amplitude is likely caused by multipath scattering of the photoacoustic signal within the near-field wire mesh, which is the same cause of acoustic clutter in the corresponding ultrasound images^52^.

Channel SNR was measured for images acquired with and without the clutter-generating wire mesh and displayed as a function of laser fluence in Fig. 1(g). The channel SNR is significantly reduced with the introduction of the clutter-generating layer, particularly at the higher fluence levels shown in Fig. 1(g). At the lowest fluence level (i.e., 6.6 mJ/cm^2^), no photoacoustic signal was present, due to the noise floor of the imaging system.

### Needle Tracking and Probe Centering

**Figure 2:**
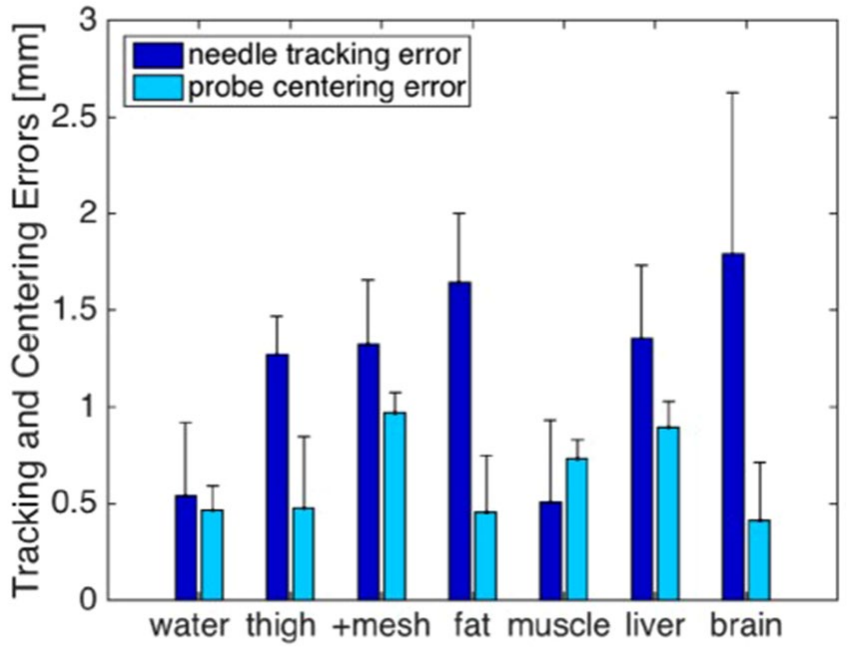
Summary of errors obtained during the needle tracking and probe centering experiments performed in water, chicken thigh (with and without the addition of the clutter-generating wire mesh), fat, muscle, liver, and brain tissues. The height of the bars represent the mean errors and the error bars show one standard deviation.

**Figure 3:**
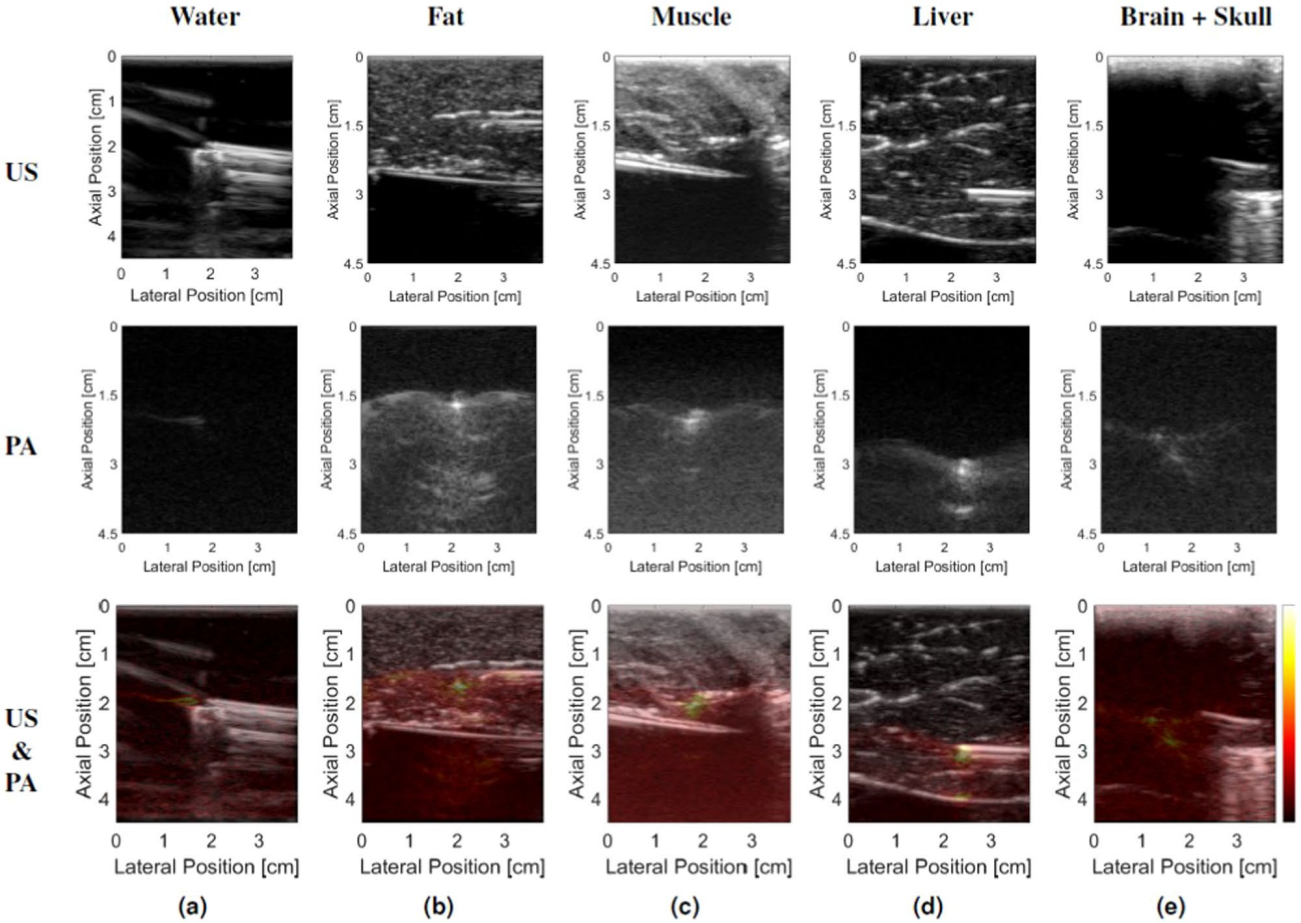
Representative ultrasound (US) and photoacoustic (PA) image pairs when visualizing the needle tip in (**a**) water, (**b**) fat, (**c**) muscle, (**d**) liver, and (**e**) brain tissue. Note that the brain tissue and inserted needle are not visible in the ultrasound image in the presence of 1 mm-thick cranial bone, yet we can detect the needle tip in the corresponding photoacoustic image. All photoacoustic images are shown with 60 dB dynamic range, with the color bar indicating the relative mapping used to represent the photoacoustic signal amplitudes in the overlaid photoacoustic images. All needle insertions angles were 0° relative to the lateral dimension of the ultrasound probe.

Results from the needle tracking and probe centering experiments are shown in Fig. 2. Representative photoacoustic images of the needle tip in the chicken thigh (with and without the clutter-generating wire mesh layer) are shown in Fig. 1, while representative photoacoustic images of the needle tip in the remaining tissue types and in water are shown in Fig. 3. It is difficult to locate the position of the needle tip in some of the ultrasound images (which is the primary motivation for a photoacoustic approach to needle tip identification). The photoacoustic images were overlaid on the ultrasound images to facilitate the identification of the needle tip in the ultrasound image. Note that the artifacts appearing below the needle tip in the photoacoustic images (e.g., in Fig. 3(b,d)) could be caused by reflections from the echogenic tissue boundaries. In the liver example (Fig. 3(d)), the combined ultrasound and photoacoustic image shows that this artifact corresponds with the tissue boundary.

Figure 2 shows that tracking errors were minimized in water and muscle. The larger tracking errors in other tissues when compared to the tracking results obtained in water indicate that the needle behaved less like a rigid body in these tissue, likely due to needle tip deflections and other factors that cause needle deformation. Tracking errors were largest in the brain tissue.

Although mean tracking errors were as large as 2 mm in some cases, they did not correlate with an increase in centering errors, as centering errors are more closely related to the robotic control accuracy, rather than the possible needle deflections that cause the needle tracking errors. A one-way ANOVA revealed no statistically significant differences in the mean centering error when the control (i.e., water) was compared to the six different tissue cases.

For each tissue type, the mean needle tracking error was <2 mm (range: 0.57–1.79 mm) with RMS errors ranging from 0.72 mm to 1.99 mm. Similarly, for each tissue type, the mean probe centering error was <1 mm (range: 0.44–0.98 mm) with RMS errors ranging from 0.53 mm to 0.99 mm. Thus, the ability of the robotic visual servoing system to center the ultrasound probe over the photoacoustic signal is within (and in most cases better than) its ability to accurately track the location of the needle tip, indicating that the probe can be centered over a needle tip with reasonable certainty in multiple tissue types.

### Angled Approach and Perturbation Recovery

**Figure 4:**
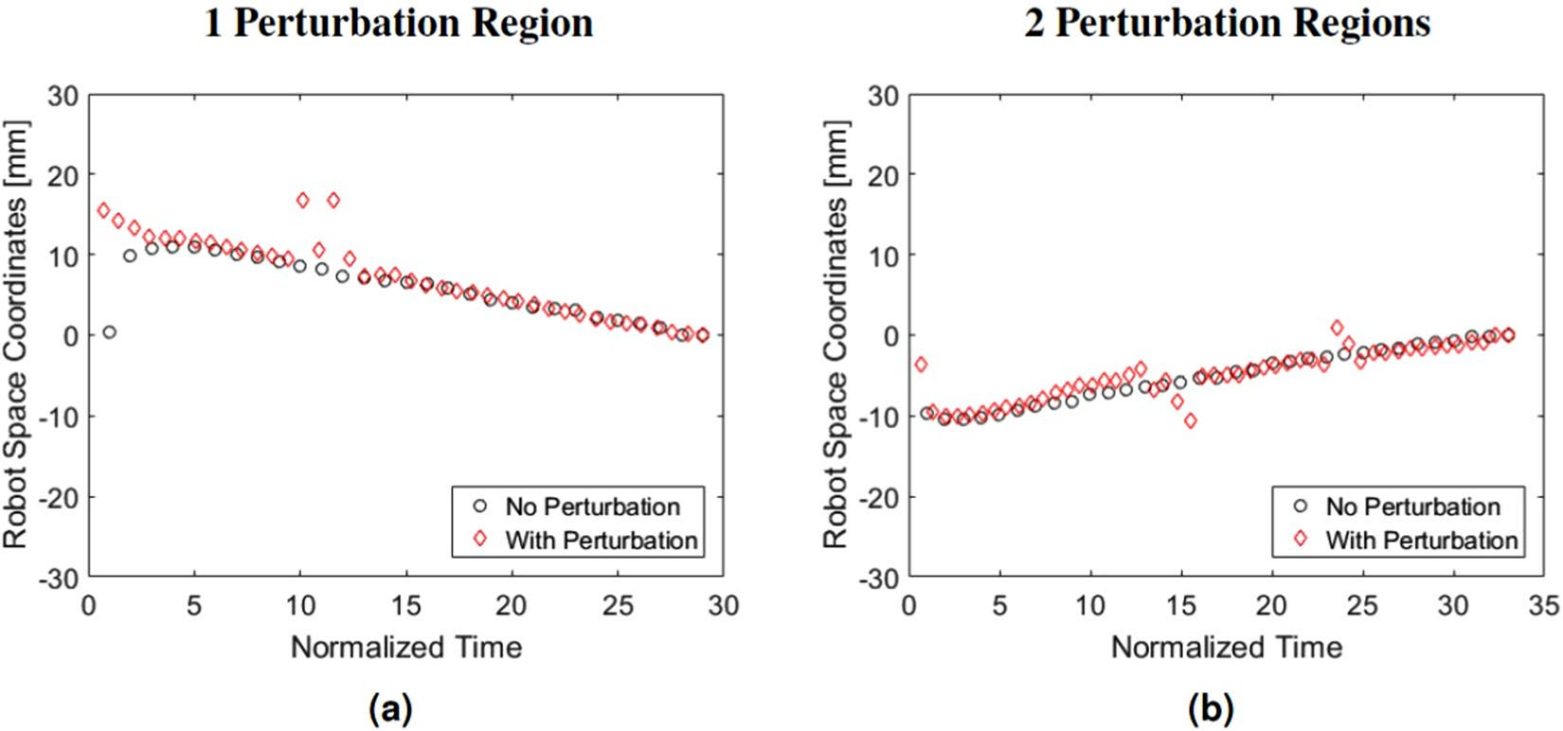
Perturbation recovery results performed with (**a**) one and (**b**) two perturbation regions. The black path shows the trajectory of the robot controlled ultrasound probe while it tracked the needle as the needle was inserted into the liver. The red path shows the same process with the addition of perturbations throughout the process where the ultrasound probe was removed from the plane of the needle tip. The robot successfully recovered from these perturbations to regain sight of the needle tip and continue tracking.

The system maintained continuous visualization of the needle tip in all 10 trials with angled needle insertions and no perturbation. For these trials without perturbation the mean centering error was 0.76 mm with one standard deviation of 0.25 mm. For the trials with perturbation the mean centering error ± one standard deviation was 0.84 mm ± 0.47 mm. Figure 4(a,b) show the trajectories of the ultrasound probe for two trials performed with the needle insertion angles of 0° and 10°, respectively. In each figure, the circles and diamonds respectively represent results in the absence and presence of perturbations applied during each trial. The x-axis time scale was normalized to obtain matching start and end times for corresponding trials performed with and without perturbation for the same insertion angle. The y-axis shows the the position of the ultrasound probe in robot space coordinates, along the primary direction of probe motion.

### Contrast and SNR Measured in Beamformed Photoacoustic Images

**Figure 5:**
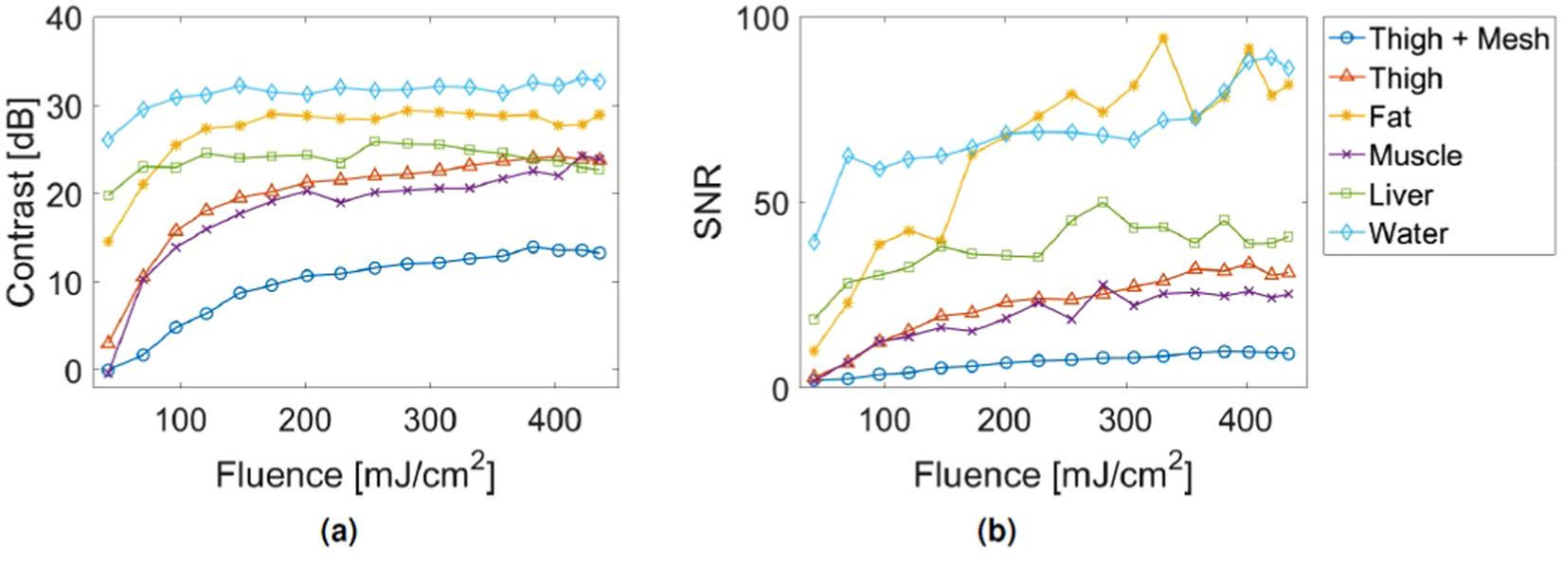
Characterization of photoacoustic image quality with regard to (**a**) Contrast and (**b**) SNR as a function of incident laser fluence. For each tissue type, similar signal contrast and SNR were obtained for a range of fluence values (including the 100 mJ/cm^2^ ANSI safety limit for a 1064 nm laser in contact with skin^53^).

The experiments with the robotic system in this manuscripts were performed with a fluence level that exceeds the 100 mJ/cm^2^ American National Standards Institute (ANSI) safety limit for a 1064 nm laser in contact with skin^53^. Corollary safety limits for specific tissues are undefined, despite the significantly different optical and thermal properties of these tissues when compared to those of skin. However, similar signals were achievable at a range of fluence values, as shown in Fig. 5. For each tissue type, similar signal contrast and SNR were obtained for a range of fluence values (including the 100 mJ/cm^2^ ANSI safety limit for a 1064 nm laser in contact with skin), indicating that the results presented above are not unique to the higher fluence levels (i.e., 201.2 mJ/cm^2^ and 350 mJ/cm^2^).

## Discussion

Our robotic approach to interventional photoacoustic imaging enabled us to track photoacoustic signals from tool tips with 0.57–1.79 mm mean accuracy and center the ultrasound probe on these photoacoustic signals with 0.44–0.98 mm mean accuracy for multiple tissue types. The sub-millimeter mean centering accuracy was obtained regardless of the tissue medium or tracking error, indicating strong potential for this system to assist with a variety of surgical procedures where traditional ultrasound image guidance fails. In particular, the tracking and centering errors are sufficiently smaller than the photoacoustic image field of view (3.84 cm in the lateral dimension), which will ensure that a needle tip (and any target of interest in front of the needle tip) will remain within the field of view of any photoacoustic image that is larger than these centering errors. In addition, the RMS tracking errors are comparble to most existing optical and electromagnetic tracking systems, which have tracking accuracies that range from 0.1 mm to 1.8 mm^54^.

While it is known that clutter causes degradation of ultrasound image quality, to the authors’ knowledge, the results in Fig. 1 are the first to demonstrate that the same clutter sources in ultrasound images impact photoacoustic images as well, manifesting as noisier channel data and reduced signal amplitudes in the beamformed photoacoustic images. We expect this effect to become more apparent with the onset of new clinical and interventional applications of photoacoustic imaging. For example, we expect similar degradation of channel SNR when the wire mesh is replaced by bone or by multiple layers of abdominal tissue. Due to the one-way sound propagation requirement for photoacoustic imaging (rather than the required two-way propagation in ultrasound imaging), the effect of acoustic clutter on photoacoustic image quality is not as significant as the same clutter levels in traditional ultrasound imaging^12^. Therefore, our results indicate that the proposed system will be successful in the presence of significant acoustic clutter in the ultrasound images.

Results additionally indicate that the proposed system will perform well in the presence of patient or operator motion that perturbs the probe location relative to the photoacoustic signal (see Fig. 4 and Supplementary Video S1), which could be caused by the operator searching for a biopsy target that is yet not located near the needle tip. This recovery from perturbations is possible due to the searching step described in the Methods section (see Perturbation Recovery and Out-of-Plane Motion Experiments). Similarly good performance is expected in the presence of reflection artifacts, similar to those appearing in the photoacoustic images of Fig. 3(b–e), due to the thresholding step described in the Methods section (see Needle Tip Segmentation). If reflection artifacts become a more significant challenge, recent deep learning approaches to photoacoustic beamforming and reflection artifact removal may assist with resolving discrepancies^55–57^, which would be particularly viable if the surgical tool is designed to produce a unique photoacoustic signature.

While a needle is used in all experiments presented in this paper, the same results could be extended to any surgical tool, such as a catheter, guide wire, or drill bit^41,58^. The primary requirement for this successful extension is that light from one or more optical fibers illuminates either the tool or surrounding tissues with sufficient energy to generate a photoacoustic signal. For example, when placing stents, photoacoustic signals could be generated either from the catheter guide wire or the blood itself and then used for intraoperative guidance and navigation. Photoacoustic catheters were previously developed for arterial wall imaging and stent placement verification^59–61^, and these catheters could be adapted for intraoperative stent guidance with an external ultrasound probe and our robotic approach.

In addition to stent placement, three other procedures that could benefit from the proposed system include percutaneous biopsy in overweight and obese patients, minimally invasive neurosurgeries, and guidance of catheters to the heart for cardiac interventions. For percutaneous biopsy, the usefulness of our proposed system would be amplified if the photoacoustic signal is used in combination with a beamformer that is tailored to difficult-to-image patients, such as the short-lag spatial coherence beamformer applied to ultrasound images^62^. Previous work demonstrated the feasibility of generating photoacoustic signals within the skull for imaging with an external ultrasound probe^39^. In addition, previous work has also demonstrated the utility of photoacoustic imaging to monitor cardiac interventions, such as the formation of radiofrequency ablation lesions or the photoacoustic-based distinction of these lesions from surrounding healthy tissue^63–65^. Therefore, incorporating the proposed robotic photoacoustic system in these four cases has promising potential to guide needles, catheters, and guide wires to the surgical site, overcome existing limitations with maintaining sight of surgical tools, and possibly reduce or eliminate fluoroscopy requirements for these procedures. The presented approach would simultaneously take advantage of the previously demonstrated benefits of photoacoustic imaging, resulting in a single imaging modality that satisfies multiple requirements for the successful completion of interventional procedures.

One possible concern for *in vivo* imaging is that strong signals from blood signal can alter the photoacoustic signal appearance and potentially affect the accuracy of the tracking results. However, to address this concern, the laser energy can be reduced without significantly affecting signal contrast and SNR (as shown in Fig. 5) to limit the size of the photoacoustic signals from blood. In addition, the segmentation step was designed to extract the coordinates of the brightest pixel in the image, based on the assumption that fluence will be greatest at the tip of the optical fiber and therefore, the brightest signal is likely to exist at the location of the fiber tip, even in the presence of blood. Alterations required to make our current system more suitable for *in vivo* use include expanding the field of view of the transducer, acquiring tip coordinates in three dimensions, and using a multi-wavelength laser with optical tuning capabilities. In addition, we did not focus on speed optimization in this paper, and the current robot control parameters would be rather slow for a surgical procedure, which is evident from Supplementary Video S1. These system modifications will be the focus of our future work.

## Conclusion

This work demonstrates visual servoing with photoacoustic imaging in five different tissue types. Through the display and analysis of channel data from acoustic environments with and without significant acoustic clutter, we additionally demonstrated that photoacoustic imaging is more robust than ultrasound imaging with regard to needle tip visualization in these challenging environments. This robustness with photoacoustic imaging is achieved because the photoacoustic signals from a single source can be coherently summed during the beamforming process, albeit with reduced contrast and channel SNR when compared to uncluttered image counterparts. In addition, our system maintained visualization of a needle tip by centering the ultrasound probe over the needle tip with sub-millimeter mean centering accuracy. This system also recovered target visualization after perturbations caused the system to lose sight of the needle tip. These results demonstrate the promise of a robotic photoacoustic system to assist with multiple interventional procedures, particularly in acoustic environments where ultrasound imaging often fails (e.g., in overweight and obese patients and in transcranial surgical applications).

## Methods

### System Overview

**Figure 6:**
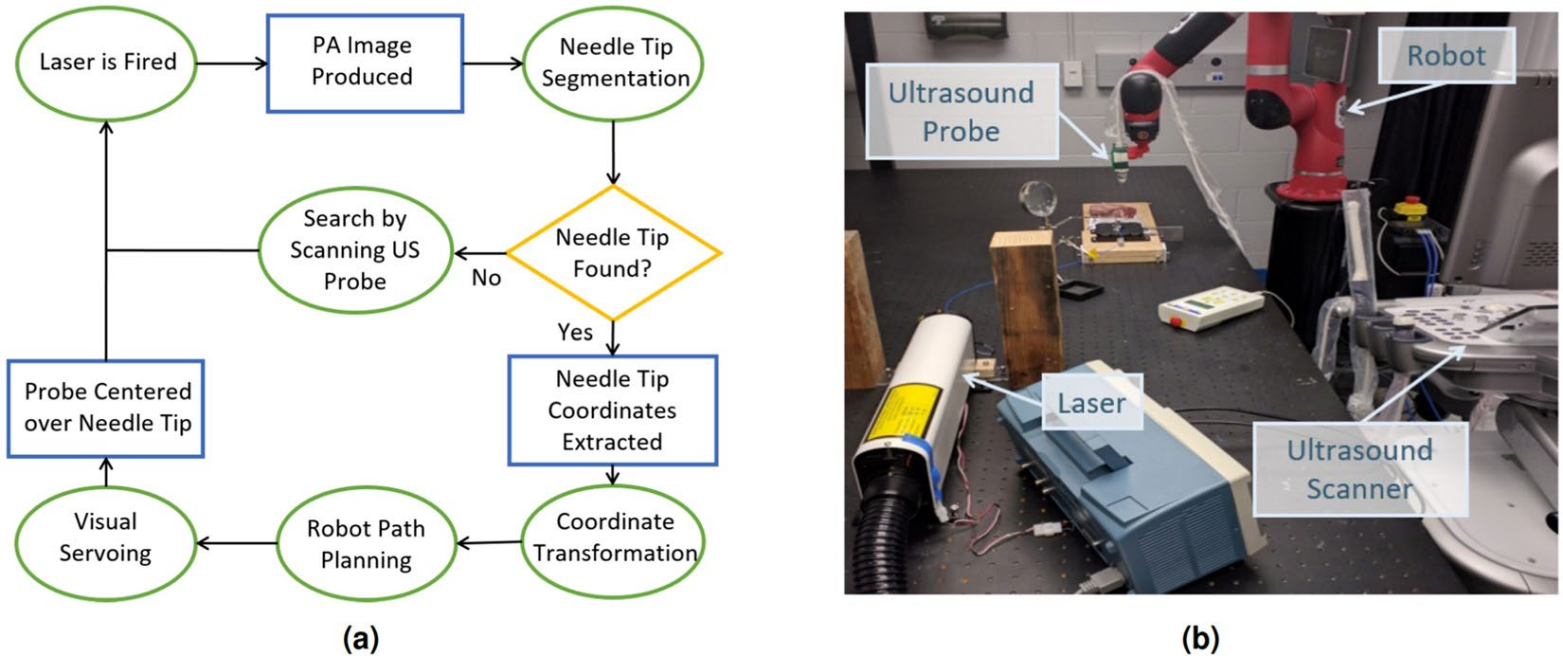
(**a**) Block diagram overview of photoacoustic visual servoing system. (**b**) Photograph of major system components. Supplementary Video S1 shows the photoacoustic-based visual servoing system maintaining sight of a needle tip being inserted in *ex vivo* bovine liver tissue, corresponding photoacoustic images, and corresponding image segmentation results that were used for robot path planning.

The general workflow of our robotic photoacoustic imaging assistant is shown in Fig. 6. The primary system components included a 1064 nm pulsed Nd:YAG laser (Opotek, Carlsbad, California, U.S.A.), an E-CUBE 12R ultrasound scanner (Alpinion Medical Systems, Seoul, Korea), and a Sawyer robot (Rethink Robotics, Boston, Massachusetts, U.S.A.). An Alpinion L3-8 linear array ultrasound transducer was attached to the end effector of the robot using a custom 3D printed probe holder. The center frequency of this transducer was 5.5 MHz and the −6 dB fractional bandwidth was 61%. The mean spatial resolution (measured as the full width at half maximum of a 0.35 mm diameter thread imaged with our photoacoustic system) was 2 mm in the lateral dimension and 1 mm in the axial dimension over the 1–3 cm photoacoustic image depths displayed in this paper. This transducer was calibrated to the robot using the method described by Kim *et al*.^66^. An optical fiber was coupled to the laser source on one end with the other end inserted into the hollow core of the needle. Unless otherwise noted, the fluence at the tip of the fiber was approximately 350 mJ/cm^2^. The ultrasound transducer (held by the Sawyer robot) was then placed onto the tissue surface and visual servoing was activated. The visual servoing system algorithm consists of needle tip segmentation followed by probe centering (described in the following two sections). Supplementary Video S1 shows the photoacoustic-based visual servoing system maintaining sight of a needle tip being inserted in *ex vivo* bovine liver tissue, corresponding photoacoustic images, and corresponding image segmentation results that were used for robot path planning. Many of the following details about our experimental methods were reported in our previous conference proceedings paper^51^ and are included below for completeness.

### Needle Tip Segmentation

To perform needle tip segmentation, binary thresholding was first applied to the photoacoustic image, with a threshold that was dynamically selected based on the maximum intensity in the image frame. Binary erosion and dilation were then performed to remove single pixel regions and increase performance of the following connected component labeling step. The pixel area was then calculated for each label and the frequency of each area measurement was displayed as a histogram. The needle tip label was selected as the label with largest area, only if the magnitude of this area was also an outlier in the histogram. If there was no distinct outlier, the algorithm assumed that the needle tip was not visible in the image frame. If an outlier existed, the needle tip location for that frame was calculated as the centroid of the labeled region. For robustness, the segmentation results from 5 previous frames were compared for spatiotemporal continuity before reporting the final decision. If there were 5 consecutive failures to find the needle tip in the photoacoustic image, the Sawyer robot scanned back and forth. At each step of the scanning, the image was segmented to locate the needle tip. If the needle tip was located, the scanning stopped. Otherwise, the scanning continued until a user-defined timeout.

### Probe Centering

If the needle tip locations, *p*, in each frame were spatially consistent, they were averaged together to produce the most likely location of the needle tip, $\bar{p}$. The vector, *p*_*center*_, was then computed as the vector from the center of the top row of the image to $\bar{p}$. The x component of this vector, ${\bar{p}}_{center,x}$, was then sent to the Sawyer robot’s control computer over a TCP/IP connection where it was then mapped into robot coordinates according to 1, where *F*_*cal*_ is the transformation obtained from calibrating the ultrasound probe and *F*_*robot*_ is the frame transformation from the robot end effector to the robot base.

1 $${p}_{robot}={F}_{robot}{F}_{cal}{\bar{p}}_{center,x}$$

A trajectory to minimize *p*_*robot*_ was then computed, resulting in the ultrasound probe being centered over $\bar{p}$.

### Experiments to Determine the Impact of Acoustic Clutter

To determine the impact of acoustic clutter on the signals generated from a needle tip, a 1 mm core diameter optical fiber was inserted through a hollow needle, and the tip of the fiber was fixed at the tip of this needle. This fiber-needle pair was fixed to a manual translation stage and inserted into an intact *ex vivo* chicken thigh.

To simulate the additional acoustic clutter that would be present in an obese patient, a water bag filled with a copper-coated wire mesh, modified from a household cleaning scrubbing pad (Scotch-Brite, 3 M Home Care Division, St. Paul, MN, U.S.A.), was placed on top of the chicken thigh, similar to implementations in previous investigations of acoustic clutter^52,67^. The scrubbing pad was unrolled, cut, and limited to two layers of the wire mesh before placing it into the water bag. The dimensions of the wire used to make the mesh are approximately 0.1 mm thickness by 0.26 mm width.

The ultrasound probe was placed on top of the wire mesh to acquire photoacoustic images, while the robot recorded the position of the ultrasound probe. With the needle tip in the same position, the wire mesh was removed from the water bag, and the imaging was repeated with the ultrasound probe in the same position. Thus, any changes in photoacoustic signal appearances were attributed to the absence of the wire mesh.

The laser energy was incrementally increased from 0.3 to 3.4 mJ, resulting in fluence that ranged from 39 $\frac{mJ}{c{m}^{2}}$ to 435 $\frac{mJ}{c{m}^{2}}$. The raw photoacoustic channel data was recorded for each fluence level. Each measurement was averaged over ten image acquisitions. The signal-to-noise ratio (SNR) of the photoacoustic channel data was computed for each image by measuring the root mean square (RMS) of the channel data (i.e., prior to beamforming).

### Needle Tracking Experiment

The fiber-needle pair was inserted into five tissue samples: (1) *ex vivo* chicken thigh (with and without the wire mesh described in the previous section), (2) *ex vivo* chicken breast (representing fat tissue), (3) *ex vivo* sirloin steak meat from the back of a cow (representing muscle tissue), (4) *ex vivo* sheep liver, and (5) *ex vivo* sheep brain tissue (imaged through an *ex vivo* human skull that was sanded to a thickness of 1 mm). Water was used as a control to obtain the best case results in the absence of needle tip deflections, optical and acoustic scattering, and acoustic clutter.

**Figure 7:**
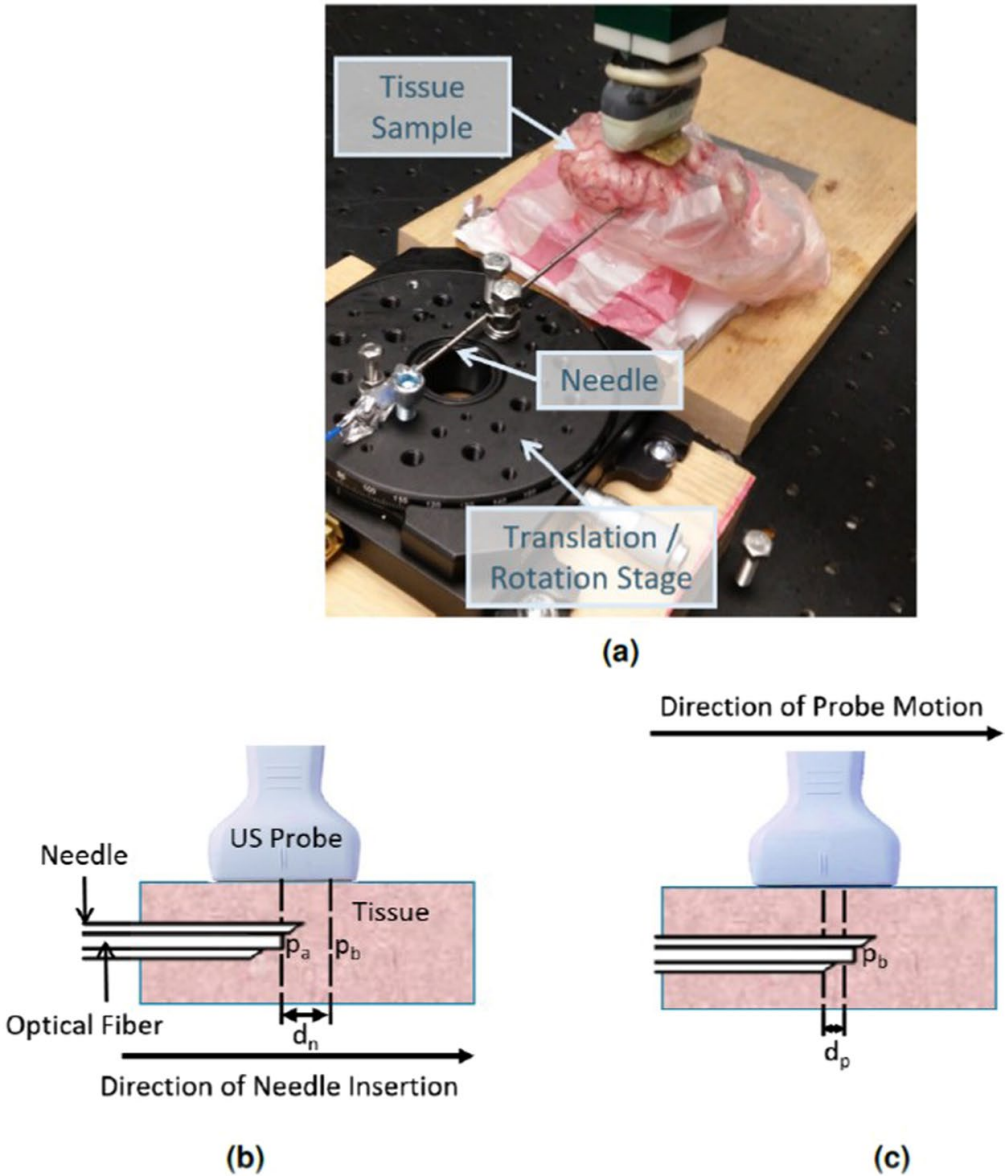
(**a**) Photograph of experimental setup showing the needle, brain tissue sample covered by skull bone, and the translation/rotation stage used to advance the needle-fiber pair into the tissue. Schematic diagrams of the (**b**) needle tracking and (**c**) probe centering experiments illustrate the measurements used to analyze experimental results: *p*_*a*_ = needle location A, *p*_*b*_ = needle location B, *d*_*n*_ = distance between *p*_*a*_ and *p*_*b*_ (recorded from image coordinates and compared to ground truth translation stage readings to report needle tracking error), *d*_*p*_ = lateral distance between the photoacoustic signal and the lateral center of the photoacoustic image after the robot stopped moving to center the probe over the needle tip (recorded from image coordinates and reported as probe centering error).

The ultrasound probe was positioned to visualize the needle tip in the photoacoustic image with the lateral axis of the ultrasound probe parallel to the needle. After this alignment was completed, the location, *p*_*a*_, of the needle tip was segmented from the photoacoustic image, and the needle was translated using a manual translation stage. The new location, *p*_*b*_, of the needle tip was then segmented from the photoacoustic image. The difference between the two needle locations in the photoacoustic image (i.e., *p*_*b*_ − *p*_*a*_) was compared to the ground truth distance, *d*_*n*_, obtained from the translation stage readings. A photograph of the experimental setup is shown in Fig. 7(a), and the distances measured for the needle tracking experiment are illustrated in Fig. 7(b).

This experiment was repeated 10 times, with the final position from the previous experiment serving as the initial position for the subsequent experiment. As a result, the needle advanced a total distance of 13 mm over the course of this experiment for each tissue type. This experiment assessed the system’s ability to estimate the correct location of the needle tip in the image.

### Probe Centering Experiment

The same set-up described in the previous section was used for the probe centering experiment, with the exception that the needle was stationary and robotic ultrasound probe movement was added. For each of the 10 trials, the probe was manually placed to visualize a needle tip that was not yet laterally centered in the image. The visual servoing software was activated and the robot moved the probe so that it became centered over the needle tip signal. The system was evaluated by measuring the lateral distance *d*_*p*_ (in image coordinates) between the photoacoustic signal and the lateral center of the photoacoustic image after the robot stopped moving, as illustrated in in Fig. 7(c). This distance was used to measure the accuracy of the robot task, which was to maintain the photoacoustic signal at the center of the image.

A one-way ANOVA was applied to the data from these probe centering experiments to determine if there were any statical differences in probe centering abilities among the various tissues.

### Perturbation Recovery and Out-of-Plane Motion Experiments

To investigate system performance when subjected to multiple insertion angles, the needle-fiber pair (fixed to the manual translation/rotation stage) was rotated from +20 to −20 degrees, in 10-degree increments, relative to the lateral axis of the ultrasound probe, with 0 degrees corresponding to the needle being parallel to the lateral axis of the ultrasound probe. Needle insertions into the liver sample were performed with these five insertion angles. The needle was continuously advanced into the liver sample a total distance of 13 mm while the robotic system segmented the needle tip location and moved the ultrasound probe to a centered position over the needle tip during this motion. If the needle tip moved out of the imaging plane of the ultrasound probe, the robotic system scanned back and forth over a distance of 60 mm in attempts to recover sight of the needle tip. If the robot found the signal, it stopped scanning and returned to centering itself over the needle tip. Otherwise, the trial would be considered a failure and the robotic system was programmed to cease visual servoing if this occurred.

The angled insertions were repeated with the addition of manual perturbation of the ultrasound probe, representative of a clinician switching to ultrasound imaging to confirm a target midway through visual servoing. The ultrasound probe was intermittently pulled away from the needle tip with cooperative control of the ultrasound probe^68^, which purposely caused the probe to lose sight of the photoacoustic signal. Then, the robot scanned the ultrasound probe back and forth over a distance of 60 mm in attempts to recover sight of the needle tip and continue visual servoing.

### Investigating the Impact of Fluence on Beamformed Photoacoustic Images

To quantify the laser fluence requirements necessary for the system to segment the needle tip from a photoacoustic image, the fiber-needle pair was inserted into four of the five tissue samples described above: (1) *ex vivo* chicken breast (representing fat), (2) *ex vivo* sirloin steak meat from the back of a cow (representing muscle), (3) *ex vivo* sheep liver, and (4) *ex vivo* chicken thigh with and without the clutter-generating wire mesh. Water was used as a control to obtain the best case results in the absence of needle tip deflections, optical and acoustic scattering, and acoustic clutter. The laser energy was incrementally increased from 0.3 to 3.4 mJ, resulting in fluence that ranged from 39 $\frac{mJ}{c{m}^{2}}$ to 435 $\frac{mJ}{c{m}^{2}}$. The raw photoacoustic channel data was recorded for each fluence level and photoacoustic images were created with delay-and-sum beamforming.

The signal contrast and signal-to-noise ratio (SNR) in beamformed photoacoustic images were computed according to the following equations:

2 $${\rm{Contrast}}=20\,{\mathrm{log}}_{10}\,(\frac{{S}_{i}}{{S}_{o}})$$

3 $$SNR=\frac{{S}_{i}}{{\sigma }_{o}}$$

where *S*_*i*_ and *S*_*o*_ are the mean of signals at the same depth inside and outside the needle tip signal (i.e., the brightest pixels in the photoacoustic image), respectively, and *σ*_*o*_ is the standard deviation of signals outside of the needle tip signal. Each measurement was averaged over ten image acquisitions.

## Electronic supplementary material

Supplementary Information File

Supplementary Video S1

## Data Availability

The data reported in this paper will be made available for non-commercial reasons upon reasonable request to the corresponding author.
