# Supplementary material for: Photoacoustic-based visual servoing of a needle tip
Source: Sci Rep. 2018 Oct 19;8:15519. doi: 10.1038/s41598-018-33931-9 (PMC6195562; doi:10.1038/s41598-018-33931-9)

Photoacoustic-based visual servoing of a needle tip: Supplementary Information

Muyinatu A. Lediju Bell[1,2,3,*] **and Joshua Shubert**[1]

[1]Johns Hopkins University, Department of Electrical and Computer Engineering, Baltimore, MD, 21218, USA
[2]Johns Hopkins University, Department of Biomedical Engineering, Baltimore, MD, 21218, USA
[3]Johns Hopkins University, Department of Computer Science, Baltimore, MD, 21218, USA
[*]mledijubell@jhu.edu

Supplementary Video S1

Supplementary Video S1 (online) shows the photoacoustic-based visual servoing system maintaining sight of a needle tip as it is inserted into *ex vivo* bovine liver tissue. Corresponding photoacoustic images and image segmentation results that were used for robot path planning are also shown in the video. Still frames from this video appear below.

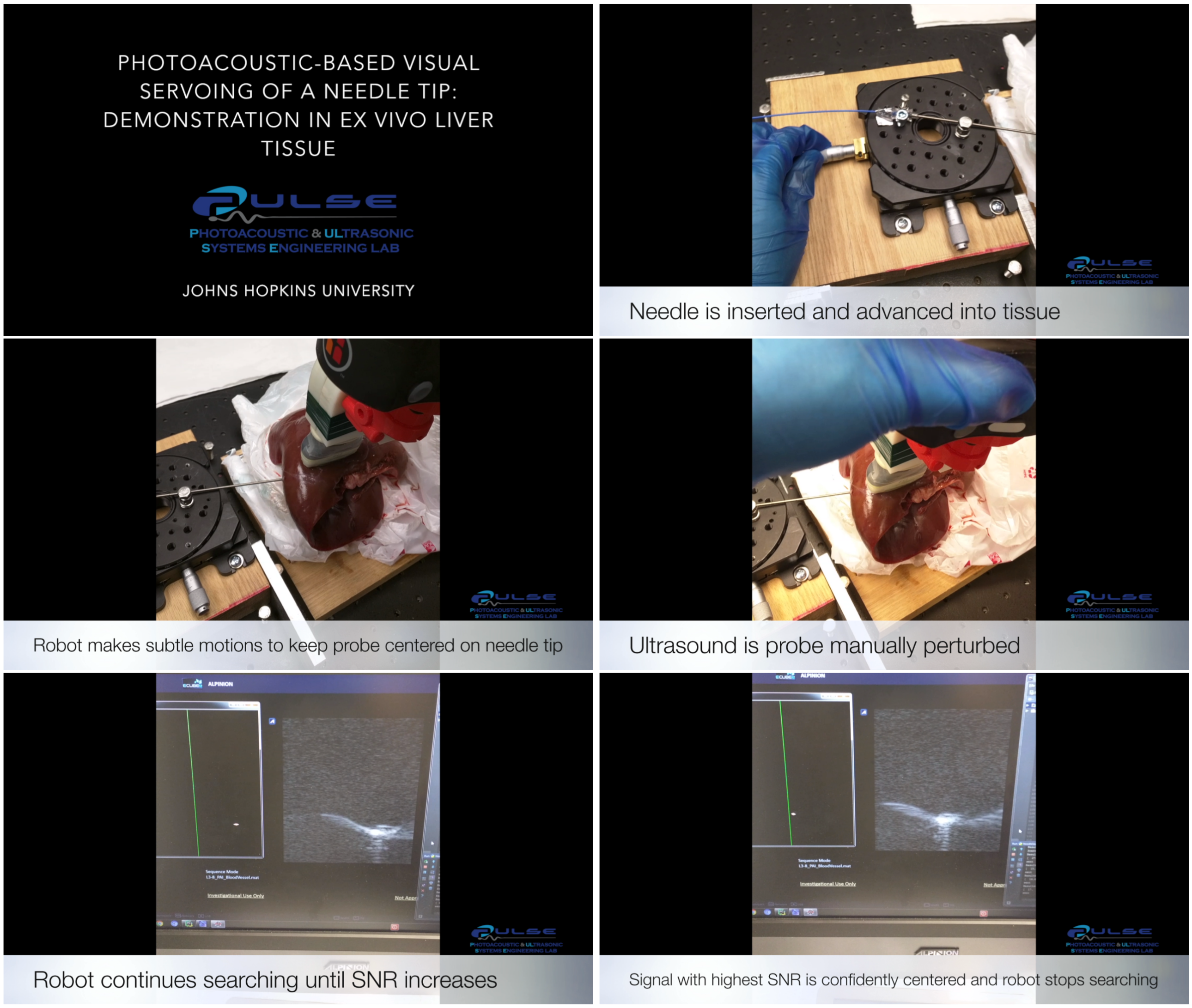

Supplement: Supplementary file 1 — Supplementary Information File [file 41598_2018_33931_MOESM1_ESM.pdf]
